# Rapid correction of severe hyponatremia and control of subsequent overcorrection in operative hysteroscopy intravascular absorption syndrome: A case report

**DOI:** 10.1097/MD.0000000000031351

**Published:** 2022-11-04

**Authors:** Seon Woo Yoo, Min-Jong Ki, Yu Jin Oh, Taehoon Kim, Seonhwa Nam, Jeongwoo Lee

**Affiliations:** a Department of Anesthesiology and Pain Medicine, Jeonbuk National University Medical School and Hospital, Jeonju, South Korea; b Research Institute of Clinical Medicine of Jeonbuk National University-Biomedical Research Institute of Jeonbuk National University Hospital, Jeonju, South Korea.

**Keywords:** 3% NaCl, desmopressin, hysteroscopic procedure, rapid correction, severe hyponatremia

## Abstract

Operative hysteroscopy intravascular absorption syndrome (OHIAS) results from systemic absorption of hypotonic solution during hysteroscopy, which may induce severe hyponatremia within hours. Depending on the serum sodium (Na^+^) level, this can be life-threatening and requires prompt and careful remedial treatment.

**Patient concerns and diagnoses:**

A 53-year-old woman underwent hysteroscopic myomectomy for submucosal leiomyoma. Approximately 3 hours postoperatively, the serum Na^+^ level decreased to 82 mM/L, accompanied by pulmonary edema and lactic acidosis. The patient was strongly suspicious of OHIAS.

**Interventions and outcomes:**

A rapid correction was made using 3% NaCl to prevent brain edema as an initial response. After the serum Na^+^ level reached 120 mM/L, gradual correction was performed considering osmotic demyelination syndrome, and desmopressin was administered to prevent overcorrection caused by excessive water diuresis. Serum Na^+^ level normalized in 4 days and the patient recovered without any specific sequelae.

**Lessons:**

The detection of OHIAS may be delayed under general anesthesia, and prior vigilance is important if the operation time is prolonged. In severe hyponatremia with an apparently rapid onset, such as OHIAS, a two-step correction process may be safe and useful: rapid correction followed by more gradual correction.

## 1. Introduction

Operative hysteroscopy intravascular absorption syndrome (OHIAS) refers to iatrogenic hypotonic hyponatremia caused by irrigation solution used in the surgical field. OHIAS is also called gynecological transurethral resection of the prostate (TURP) syndrome due to its similarity to TURP syndrome in pathogenesis, clinical picture, and treatment. It can cause seizures and respiratory arrest in postoperative patients, progressing rapidly to death or a permanent vegetative state.^[1]^ Although early detection is important, even obstetricians and anesthesiologists are often unaware of OHIAS. Once hyponatremia has occurred, it is important to determine the correction rate. A sharp overcorrection of serum Na^+^ level increases the risk of osmotic demyelinating syndrome (ODS). In contrast, life-threatening cerebral edema can occur if serum Na^+^ level does not rise to the target range within a period.^[2,3]^

Here, we report the case of a female patient with a serum Na^+^ level of 82 mM/L after hysteroscopic surgery. It is a rare case where the sodium level suddenly dropped iatrogenically within 3 hours after surgery, and we performed a two-step correction with reference to several guidelines. Furthermore, we discuss the adopted initial correction rate and target values to minimize neurological sequelae and the subsequent occurrence of diluted urine and overcorrection.

## 2. Case presentation

Informed consent was obtained from the patient for this study. A 53-year-old woman weighing 60 kg and 152 cm in height was diagnosed with submucosal leiomyoma. She decided to undergo a hysteroscopic myomectomy at the gynecology department of our hospital. She had been taking medications for hypertension and intermittent nonspecific angina.

Preoperative blood test results showed no abnormalities, and all electrolytes were within normal ranges (Na^+^, K^+^, and Cl^–^ were 139, 4.1, and 106 mM/L, respectively). Furthermore, there were no abnormal findings on preoperative electrocardiography or echocardiography.

On the day of surgery, electrocardiogram, pulse oximetry, blood pressure, and body temperature were measured according to routine procedures in the operating room. Her blood pressure, heart rate, respiratory rate, and oxygen saturation were 136/84 mm Hg, 106 bpm, 15 breaths/minute, and 96%, respectively. Urosol (HK inno.N, Seoul, South Korea), a hypotonic solution (190 mOsm) containing 540 mg of D-mannitol and 2.7 g of D-sorbitol per liter, was used as a distension medium for the hysteroscopic surgery.^[4]^ Two hours after initiation of surgery, arterial blood gas analysis showed severe hyponatremia (Na^+^ <100 mM/L), as well as acidosis (pH 7.22, lactate 2.7 mM/L), hypocalcemia (Ca^2+^ 0.75 mM/L), hypoglycemia (glucose 74 mg/dL), and anemia (hematocrit 19%). The total surgery time was 2 h 50 minute, during which 45 L of distension medium was used (15 × 3 L Urosol bags), and total urine output was 2400 mL.

**Figure 1.**
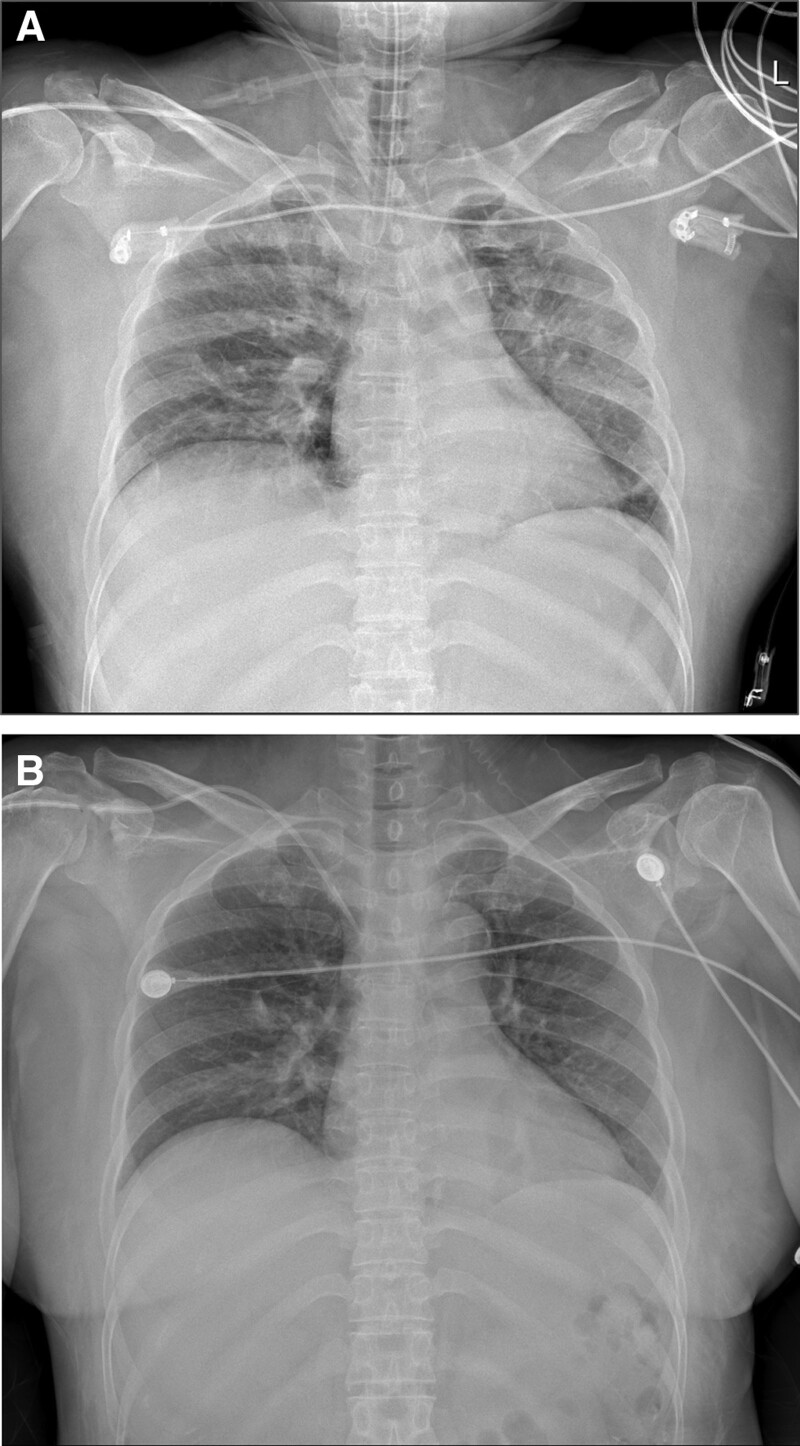
Comparison of anteroposterior chest radiographs immediately after transfer to the surgical intensive care unit (A) and 10 h post-surgery (B).

The patient was transferred to the surgical intensive care unit while maintaining intubation without emergence from anesthesia. Initial postoperative laboratory tests showed severe hyponatremia, with Na^+^, K^+^, and Cl^–^ levels of 82, 4.4, and 62 mM/L, respectively. Lactate level increased to 8.7 mM/L. An anteroposterior chest radiograph revealed pulmonary edema in both the lungs (Fig. 1A). Serum osmolarity, urine osmolarity, urine Na^+^, and urine creatinine levels were 246 mOsm/kg (reference values: >275 and < 295 mOsm/kg), 289 mOsm/kg (reference values: >400 and < 800 mOsm/kg), 4 mM/L and 2.98 mg/dL (reference values: >90 and < 300 mOsm/kg), respectively. The patient was strongly suspicious of OHIAS.

**Figure 2.**
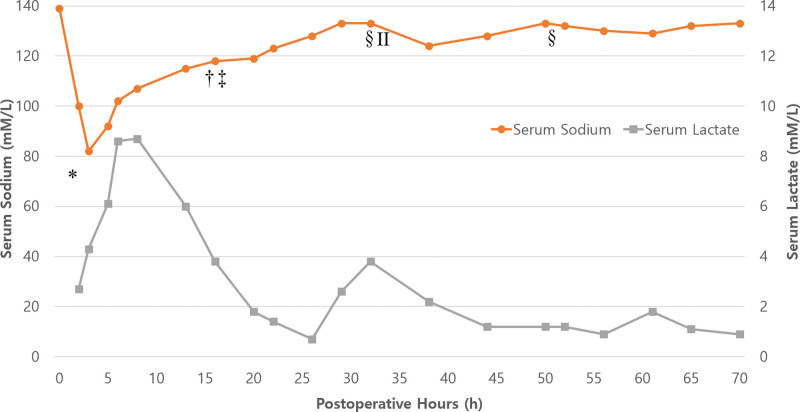
Graph representing fluctuations in serum sodium (mM/L) and serum lactate (mM/L) over time (h). * Admission to the surgical intensive care unit. † Main fluid changed from 3% to 0.9% NaCl. ‡ Extubation. § Desmopressin (2 µg) was administered intravenously. ^ΙΙ^ Main fluid changes from 0.9% NaCl to 5% dextrose.

In response, we promptly decided to perform rapid correction with intravenous administration of 3% NaCl, starting at 100 ml/h. The serum Na^+^ levels after approximately 1 and 3 h were 92 and 102 mM/L, respectively. Accordingly, the administration rate was lowered to 50 mL/h, and serum Na^+^ level was checked every 2 h and adjusted to 10–50 mL/h. Approximately 12 h after correction, the serum Na^+^ levels were 118 mM/L (Fig. 2), and 3% NaCl was replaced with 0.9% NaCl. Pulmonary edema, observed on chest radiography, also improved (Fig. 1B), and the lactate levels were normalized from 8.7 to 1.8 mM/L.

The patient consistently excreted large amounts of dilute urine. During the first 10 h of transfer to the surgical intensive care unit, the fluid intake (2050 mL) and output (5040 mL) difference (I/O) was –2990 mL. The 24-h I/O measured the next day was –4190 mL (intake, 6770 mL; output, 10,960 mL). This caused the serum Na^+^ level to increase more rapidly than predicted. The serum Na^+^ level rapidly increased from 118 mM/L to 133 mM/L within 12 hours even after stopping the administration of 3% NaCl. Subsequently, we administered two doses of 2 µg desmopressin (Minirin® 4 µg/mL ampules, Ferring Pharmaceuticals, Malmo, Sweden). Consequently, serum Na^+^ level elevation was somewhat stabilized, and urine output decreased (I/O on postoperative day [POD] 3 and 4 were –2630 and –1745 mL, respectively). Gradually, urine concentrations of Na^+^ and creatinine increased to 25 mM/L and 40.51 mg/dL, respectively. The serum Na^+^ level increased steadily as planned and normalized at 4 days postoperatively (135 mM/L).

The patient was transferred to the general ward on POD 4 and discharged on POD 8. Enhanced magnetic resonance imaging and angiography conducted on POD 7 revealed no remarkable abnormalities in the brain parenchyma, ventricular system, or extracerebral cerebrospinal fluid space. The patient complained of a mild headache after discharge and followed-up with a neurologist for consultation. The headache improved three months after discharge, and no other sequelae were reported.

## 3. Discussion

OHIAS is caused by systemic absorption of excessive irrigation solution in a closed space during hysteroscopic surgery. The irrigation solution used is typically a hypotonic, non-conductive fluid to avoid thermal burn. When absorbed systemically, it induces hyponatremia in proportion to the amount used. The major risk factors are use of large amounts of hypotonic fluid, prolonged operation time, operative procedures (not diagnostic), exposure of uterine venous sinus, and high irrigation fluid pressure (>10 mm Hg). In adult women, it has been reported that when 1000 ml of non-conductive solution is absorbed, there is a decrease of serum sodium of 10 mEq/L and that for 3000 ml is 30 mEq/L.^[4]^ This suggests that sufficient attention should be paid to severe hyponatremia (serum Na^+^ <120 mm Hg) when using more than 2000 ml of irrigation solution. In our patient, 4500 ml of irrigation solution was used for 2 hours and 50 minutes of myomectomy.

Early detection and intervention are important for positive outcomes in cases of OHIAS; however, detection may be difficult or nearly impossible while the patient is under general anesthesia and cannot complain of symptoms. In severe hyponatremia, tachycardia, hypotension, and high airway pressure may occur, but they can be easily overlooked without careful attention. In our case, there was no significant change in vital signs or airway pressure, but a large amount of urine output (1800 mL) was the only clue to the impression.

After confirming that the serum Na^+^ level was 82 mM/L, we planned a two-step calibration. The initial goal was rapid correction to 120 mM; then adjustment would proceed gradually to < 8 mM/day. Based on the above, we reduced the possibility of cerebral edema leading to brain herniation by aiming to reach a Na^+^ level of 120 mM/L within the first 24 h and then minimized the likelihood of ODS by performing subsequent gradual correction.

The US guidelines recommend 100 mL of 3% NaCl intravenous infusion over 10 minure 3 times as needed without limitation of the rate of correction in true acute hyponatremia. Patients with hyponatremia for less than 24 hours are highly unlikely to develop ODS.^[5]^ Among other things, brain herniation, the most dangerous complication of hyponatremia, occurs almost exclusively in patients with acute hyponatremia.^[6]^ In the majority of OHIAS and TURP syndrome cases, a sharp decrease in serum Na^+^ level by electrolyte-free lavage did occur but was successfully reversed.^[7]^ In patients with severe postoperative hyponatremia (80–110 mM), use of 3% NaCl solution could increase serum Na^+^ level by 20–30 mM within 12–24 hours, and most of them were successfully treated without sequelae. This is supported by the fact that the rapid onset of hyponatremia is so short-lived that cerebral adaptation is insufficient; therefore, the risk of ODS is low.^[5]^ On the other hand, a relatively slow correction could potentially promote the risk of ODS by allowing sufficient time for cerebral adaptation.^[3]^

Nevertheless, European and American colleges of obstetricians and gynecologists guidelines still recommend not exceeding 10 mEq/L and 12 mEq/L, respectively, during the first 24 hours. Although the US guidelines are more liberal, they also warn of the dangers of ODS for correction above 12 mM/L in the first 24 h.^[8,9]^ Rapid correction is probably safe, but the risk of ODS is not completely ruled out. They suggest that an increase of 4–6 mM/L for the first few hours is sufficient to alleviate the symptoms and prevent brain herniation.

The increase in serum Na^+^ level after administration of hypertonic saline may be much higher than expected from the formula.^[10]^ In this case, the Na^+^ concentration increased from 82 to 118 mM/L during the first 12 h, and the required 3% NaCl concentration was calculated to be approximately 2105 mL using the classic formula. However, The actual amount of 3% saline administered to increase the Na^+^ level by 36 mM/L for this patient was approximately 400 mL (19% of the calculated amount), not 2105 mL. The patient was in a severe state, with excessive body fluid and a large amount of diluted urine (5400 mL) was simultaneously excreted.

After the serum Na^+^ level reached 120 mM/L, 3% saline was replaced with normal saline for gradual correction, but the unexpected increase continued. Desmopressin effectively controlled the overcorrection that occurred rapidly during hyponatremia correction because of the excretion of large amount of diluted urine. Desmopressin (2 µg) was administered twice, and the 6-h urine output before and after administration rapidly decreased from 4100 to 1220 mL and from 2680 to 380 mL, respectively. The US and European guidelines recommend parenteral administration of 2–-4 µg of desmopressin to prevent water diuresis when overcorrection occurs, with repeated dosing every 8 hours based on a serial Na^+^ check. In addition, appropriate desmopressin can make hyponatremia correction more predictable by reducing excessive urine water.^[11–13]^

## 4. Conclusion

The patient’s lowest serum sodium concentration and duration of hyponatremia should be considered in determining the correction rate. Although no consensus has been reached regarding the ideal initial correction rate, rapid correction will likely be safe in the event of obviously sharp hyponatremia, and the potential benefits outweigh the risks. Further studies related to ODS and brain adaptation time are needed, and based on this, a more detailed guideline on treatment strategies for iatrogenic acute severe hyponatremia such as OHIAS is required.

## Author contributions

**Conceptualization:** Jeongwoo Lee.

**Data curation:** Yu Jin Oh.

**Formal analysis:** Taehoon Kim.

**Visualization:** Seonhwa Nam.

**Writing – original draft:** Seon Woo Yoo.

**Writing – review & editing:** Jeongwoo Lee, Min-Jong Ki.

## Acknowledgements

We would like to express our gratitude to the professors of nephrology and gynecology for their generous advice regarding the treatment of patients. In addition, this manuscript has been edited in English with the help of Editage.

## References

[1] Giuliani C Peri A. Effects of hyponatremia on the brain. J Clin Med. 2014;3:1163–77. 26237597 10.3390/jcm3041163 PMC4470176

[2] Sterns RH. Treatment of severe hyponatremia. Clin J Am Soc Nephrol. 2018;13:641–9. 29295830 10.2215/CJN.10440917 PMC5968908

[3] Adrogué HJ Madias NE. Hyponatremia. N Engl J Med. 2000;342:1581–9. 10824078 10.1056/NEJM200005253422107

[4] Istre O Skajaa K Schjoensby AP. Changes in serum electrolytes after transcervical resection of endometrium and submucous fibroids with use of glycine 1.5% for uterine irrigation. Obstet Gynecol. 1992;80:218–22. 1635735

[5] Verbalis JG Goldsmith SR Greenberg A. Diagnosis, evaluation, and treatment of hyponatremia: expert panel recommendations. Am J Med. 2013;126(suppl 1):S1–42. 10.1016/j.amjmed.2013.07.006 24074529

[6] Gankam Kengne F Decaux G. Hyponatremia and the brain. Kidney Int Rep. 2018;3:24–35. 29340311 10.1016/j.ekir.2017.08.015 PMC5762960

[7] Atieh AS Abu Shamma OK Abdelhafez MO. Acute severe hyponatremia following hysteroscopic procedure in a young patient: a case report and review of the literature. Case Rep Nephrol. 2021;2021:7195660. 34594582 10.1155/2021/7195660 PMC8478601

[8] Spasovski G Vanholder R Allolio B. Clinical practice guideline on diagnosis and treatment of hyponatraemia. Eur J Endocrinol. 2014;170:G1–47. 24569125 10.1530/EJE-13-1020

[9] American College of Obstetricians and Gynecologists. ACOG technology assessment in obstetrics and gynecology. Obstet Gynecol. 2005;106:439–442. 16055609 10.1097/00006250-200508000-00054

[10] Mohmand HK Issa D Ahmad Z. Hypertonic saline for hyponatremia: risk of inadvertent overcorrection. Clin J Am Soc Nephrol. 2007;2:1110–7. 17913972 10.2215/CJN.00910207

[11] Perianayagam A Sterns RH Silver SM. DDAVP is effective in preventing and reversing inadvertent overcorrection of hyponatremia. Clin J Am Soc Nephrol. 2008;3:331–6. 18235152 10.2215/CJN.03190807 PMC2390955

[12] MacMillan TE Tang T Cavalcanti RB. Desmopressin to prevent rapid sodium correction in severe hyponatremia: a systematic review. Am J Med. 2015;128:1362.e15–24. 10.1016/j.amjmed.2015.04.040 26031887

[13] Ward FL Tobe SW Naimark DMJ. The role of desmopressin in the management of severe, hypovolemic hyponatremia: a single-center, comparative analysis. Can J Kidney Health Dis. 2018;5:2054358118761051. 10.1177/2054358118761051 PMC5865454 29593879

